# Epigenetic reprogramming in liver fibrosis and cancer^☆^

**DOI:** 10.1016/j.addr.2017.10.011

**Published:** 2017-11-01

**Authors:** Caroline L. Wilson, Derek A. Mann, Lee A. Borthwick

**Affiliations:** Fibrosis Research Group, Institute of Cellular Medicine, Newcastle University, UK

**Keywords:** Hepatic Stellate Cell, Hepatocellular carcinoma, DNA methylation, Histone modifications, Non-coding RNAs

## Abstract

Novel insights into the epigenetic control of chronic liver diseases are now emerging. Recent advances in our understanding of the critical roles of DNA methylation, histone modifications and ncRNA may now be exploited to improve management of fibrosis/cirrhosis and cancer. Furthermore, improved technologies for the detection of epigenetic markers from patients' blood and tissues will vastly improve diagnosis, treatment options and prognostic tracking. The aim of this review is to present recent findings from the field of liver epigenetics and to explore their potential for translation into therapeutics to prevent disease promoting epigenome reprogramming and reverse epigenetic changes.

## Graphical abstract

**Figure:**
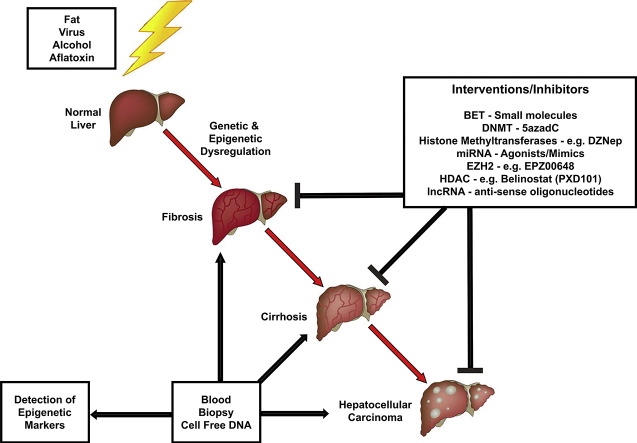

## 1 Introduction

Epigenetics is the study of how the genome is interpreted by the cell to generate a phenotype. If taken literally this could include all of the events involved in the regulation of gene expression. However for the purpose of this review discussion will be limited to those regulatory mechanisms that operate at the level of DNA methylation, histone modifications and the activities on non-coding regulatory RNA molecules. But in addition, readers should be aware of the critical role that transcription factors play in determining gene expression and cell phenotype. Transcription factors, which operate by recognising specific DNA motifs in the promoter/enhancer regions of genes and then modulate the activity of RNA polymerase II, are increasingly featuring as important drug targets in chronic liver disease. Typical examples being the peroxisome proliferator-activated receptors and the farnesoid X receptor for which ongoing clinical trials are determining efficacy in non-alcoholic fatty liver disease (NAFLD) and autoimmune liver disease [1], [2], [3].

DNA methylation is a direct chemical modification of DNA in which methyl groups are predominantly added to cytosine residues within the context of a CpG dinucleotide [4]. This modification is carried out by three highly conserved enzymes, DNA methyltransferase 1 (DNMT1), which maintains the 5me-CpG mark during cell divisions, and the DNMT3A and DNMT3B enzymes that function as *de novo* methyltransferases, establishing methylation patterns during development [5]. As a general rule the 5me-CpG mark is associated with gene repression and in particular when it is associated with CpG-rich promoter regions [6]. Hence, high density CpG promoters that are typical of housekeeping genes are rarely methylated, while genes that have intermediate density CpG content are silenced upon methylation [7], [8]. In contrast low density CpG rich promoters remain transcriptionally active even when hypermethylated. However, this perhaps over simplistic rule breaks down in other regions of the genome, in particular within gene bodies where DNA methylation can be associated with active transcription [9]. Discovery of the three ten eleven translocation (TET1-3) enzymes, which catalyse progressive oxidation of the 5-meCpG mark, implies that DNA methylation is more dynamic than previously thought [10]. Disturbance in the balance of DNMT and TET activities has potential to contribute to disease progression as evidenced by mutations in DNMT3A and TET2 being frequently found in human cancers [11].

DNA is packaged into chromatin of which the unit structure is the nucleosome which is comprised of two copies each of histones H2A, H2B, H3 and H4 that assemble into an octamer around which 146–147 bp of DNA is tightly wrapped. The nucleosome is a highly dynamic structure and dictates the degree to which DNA is accessible for transcription; this being determined by its degree of compaction and by post-translational modifications (PTMs) on the N-terminal tails of its constituent histones [12]. The core histone tails can be modified by acetylation, methylation, ubiquitination, sumoylation and phosphorylation which combine to regulate chromatin structure and gene expression. Lysine acetylation and methylation are the best characterised histone PTMs, with the former being associated with transcriptionally active genes while lysine methylation has a more modulatory function with its influence determined by the location of the lysine residue on the histone tail and the extent of its methylation (mono, di or trimethylation). Acetylation is regulated by histone acetyltransferases (HATs) and histone deacetylases (HDACs). HDACs are targets for an expanding catalogue of drugs many of which are in clinical studies in human cancers where dysregulation of histone acetylation is mechanistically implicated in dysregulation of gene expression [13]. Histone lysine methyltransferases (HMTs) and demethylases (KDMs) are also implicated in human disease and are consequently subject to intense drug discovery [14]. Histone PTMs function as recognition signals for so-called histone modification readers, these being nuclear proteins that transmit the structural information in the chromatin to the transcriptional machinery. Bromodomain-containing proteins or BETs (e.g. BRD2, BRD3, BRD4, BRDT, ASH1L) are a particularly important class of histone readers that recognise acetylated lysine residues and are key players in cancer and inflammation [15]. Small molecule BET inhibitors have shown great pre-clinical promise and are now in a variety of clinical studies [16].

The vast majority of the human genome is transcribed, however only 2% encodes proteins. The vast majority of the transcriptome consists of non-coding RNAs (ncRNAs) that have regulatory functions and include micro RNAs (miRNA), small nucleolar RNAs (snoRNAs), small interfering RNAs (siRNAs), Piwi-interacting RNAs (piRNAs) and the long non-coding RNAs (lncRNAs). The miRNAs are an extensively studied class of single-stranded 18–22 nucleotide ncRNAs that fine-tune expression of the genome either by decreasing the stability or suppressing translation of messenger RNAs [17]. The physiological importance of miRNAs is demonstrated by the evidence for genetic and epigenetic alterations in miRNA biogenesis being associated with oncogenesis [18]. Moreover, miRNAs can be specifically targeted by chemically modified antisense oligonucleotides which raises potential for their therapeutic manipulation [19]. Recently lncRNAs, a large and diverse class of transcribed RNA molecules with a length of > 200 nucleotides that do not encode proteins, have attracted considerable attention because of their myriad of functions including control of chromatin remodelling, gene transcription, protein transport and metabolism [20], [21]. Mutations and dysregulated expression of lncRNAs are associated with a variety of diseases including diabetes and cancer, raising potential for their application as biomarkers for disease onset and progression [22].

Here we will consider recent advances in the role of DNA methylation, histone modifications and ncRNAs in chronic liver diseases and in particular how new knowledge of these epigenetic mechanisms may be exploited for improving the management of the two major liver disease endpoints, fibrosis/cirrhosis and cancer.

## 2 Environmental impacts on the hepatic epigenetic landscape

The liver must adapt on a daily basis to a constant flux of environmental variations including circadian oscillators, nutritional/metabolic fluxes, exposure to xenobiotics, viral infections, alterations in the microbiome and the demands for epithelial repair and regeneration. Using a deep sequencing approach, Vollmers and colleagues systematically mapped the epigenetic changes occurring over 24 h in the mouse liver [23]. Temporal changes were observed in 1262 transcripts of which 464 were identified as protein coding, 19 as lncRNAs and 53 as miRNAs including Let-7, miR-33, miR-103 and miR-122 that have previously been associated with functional roles in liver physiology and diseases [24], [25], [26], [27]. Integrated with these transcriptional changes were genome-wide oscillations in histone lysine modifications including for the mark of transcriptional active chromatin H3K4me3, found at 826 gene promoters. Noteworthy was the absence of changes in DNA methylation indicating the stability of this epigenetic mark in the normal healthy liver. By contrast profound changes in DNA methylation occur in the livers of mice fed a lipogenic diet that induces steatosis, an effect associated with altered expression of DNMT1 and DNMT3A [28]. In humans, obesity has been associated with accelerated liver ageing based on analysis of the DNA methylome [29] and in human Nonalcoholic steatohepatitis (NASH) increased DNMT1 is reported [30].

HDAC3 is an important circadian regulated epigenetic writer critical for hepatic triglyceride homoeostasis and when deleted in mice results in steatosis, inflammation and fibrosis [31], [32]. Similarly the HDAC, SIRT1 prevents NAFLD by regulating adipogenesis and suppressing NF-κB-driven inflammation [33]. Around 100 miRNAs are dysregulated in human NASH including the circadian regulated miR-122 which is under-expressed in NASH [34]. Alcohol depletes S-adenosylmethionine (SAMe), the major methyl donor for DNA and histone methylation, alters the expression of multiple miRNAs and *via* its induction of hepatic reactive oxygen species (ROS) promotes H3K9 acetylation leading to increased expression of alcohol-metabolising enzymes that induce further ROS and acetylation potentially establishing feed-forward epigenetic re-landscaping [35].

Hepatitis C virus (HCV) infection promotes multiple changes in DNA methylation including at enhancer elements associated with altered expression of genes implicated in cancer and control of stem cells [36]. The hepatitis B virus X protein (HBx) also alters DNA methylation *via* its ability to influence DNMT activity and in addition recruits the histone acetyltransferases p300/CBP to induce IL-8 and proliferating cell nuclear antigen (PCNA) which are involved in inflammation and cell proliferation respectively [37]. Recent work by Thaiss et al. has shown that the gut microbiota exhibits a diurnal rhythmicity that programs in a circadian manner the liver transcriptome and its detoxification pattern [38]. As the gut microbiota is dramatically modified in alcoholic and non-alcoholic liver diseases this new discovery has major implications for epigenetic control of oscillatory hepatic gene expression and liver function [39], [40].

Hence, in summary the epigenetic landscape of the liver is acutely sensitive to environmental cues resulting in modifications that impact on its circadian controlled patterns of gene expression. In obesity and alcoholic disease the disturbance to the hepatic epigenetic landscape may promote microenvironments in which steatosis advances to inflammation, fibrosis and cancer.

## 3 Epigenetic drivers of liver fibrosis

Myofibroblasts are the major cellular drivers of liver fibrosis and appear during conditions of liver damage and/or infection mainly through the process of hepatic stellate cell (HSC) transdifferentiation. The conversion of quiescent HSC to their activated myofibroblast state involves vast changes in transcriptome expression that dramatically alter the phenotype and behaviour of the cell. In the past decade we have begun to shed light on the epigenetic events that reprogram the HSC transcriptome, thus revealing new regulators of fibrogenesis as well as potential biomarkers for tracking disease progression.

### 3.1 DNA methylation

**Fig. 1:**
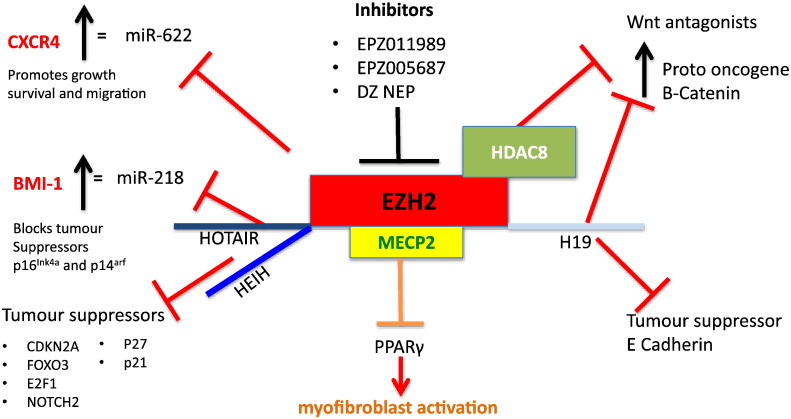
The Implications of EZH2 as a therapeutic target in fibrosis and HCC. The schematic outlines some of the ways in which EZH2 is able to support growth, survival and migration of liver cancer and activation of the scar forming myofibroblasts of the liver.

Many of the regulatory events associated with *in-vivo* transdifferentiation of HSC can be recapitulated in a cell culture model in which isolated HSC are maintained for several days on plastic in serum-containing media. In this widely used model, HSC transdifferentiation was accompanied by a > 20% methylation change (hypo- or hyper-methylation) in ~ 400 methylated regions including DNMT3A and DNMT3B [41]. These changes in methylation were associated with either transcriptional repression or activation. For example, peroxisome proliferator-activated receptor γ (PPARγ) gene silencing is required for HSC activation [42] and this process is regulated by two concurrent methylation-based epigenetic control mechanisms. Firstly, the DNA methylation reader MeCP2 is recruited to methyl-CpGs located in the promotor region of PPARγ and subsequently directs repressive H3K9me3-modifying enzymes to suppress initiation of transcription. Secondly, transcriptional elongation is suppressed by EZH2-mediated H3K27me3 modifications in the downstream coding region of PPARγ, with expression of EZH2 being dependent on MeCP2 [43] (Fig. 1). Significantly, HSC isolated from mice deficient in MeCP2 show attenuated levels of classical myofibroblasts markers such as α-smooth muscle actin (αSMA) or collagen 1 and are protected from carbon tetrachloride (CCl_4_) induced liver fibrosis [43]. Conversely, MeCP2 has also been shown to stimulate the transcription of multiple pro-fibrotic genes through the control of ASH1, an H3K4/H3K36 histone methyltransferase that directly binds to the regulatory regions of αSMA, collagen 1, TIMP1 and TGF-β1 in activated HSCs, promoting their transcription [44]. Jumonji Domain-Containing Protein 1A (JMJD1A), an H3K9 demethylase involved in adipogenic metabolism, was also found to regulate PPARγ gene expression. Specifically, knockdown of JMJD1A in HSC correlated with reinforced H3K9me2 in the PPARγ gene promoter, increased αSMA and collagen expression, and enhanced necrosis in the CCl_4_ mouse fibrosis model [45]. Taken together the data suggests that MeCP2 and JMJD1A are critical epigenetic regulators of HSC phenotype and fibrogenesis.

DNA methylation in the PPARγ gene promotor has also been shown to be relevant in human disease. In a well phenotyped cohort of patients with biopsy proven NAFLD Zeybel et al. demonstrated that hypermethylation at discreet CpG dinucleotides within the human PPARγ gene promotor could be used to stratify patients with mild fibrosis (Kleiner score 0–2) from those with severe fibrosis (Kleiner score 3–4) [46], [47]. In a similar study in a cohort of Hepatitis B virus (HBV) infected patients, disease progression correlated with DNA methylation changes at CpG dinucleotides in the HoxA2, PPP1R18 and HDAC4 genes [47]. However, measurements of DNA methylation in these studies relied on access to liver biopsy tissue. More recently this limitation was overcome by using pyrosequencing to quantify differential DNA methylation at the PPARγ promoter from circulating cell-free DNA extracted from patient plasma. Again this method was able to stratify between mild and severe fibrosis in NAFLD [48]. If validated, this exciting approach could be utilised as a potential plasma biomarker of liver fibrosis progression that negates the need for biopsy. Furthermore, this study proposed that the source of circulating cell-free DNA is likely to be dying/damaged hepatocytes, this interesting finding suggests that fibrosis progression is accompanied (or driven) by reprogramming of the hepatocyte DNA methylome.

As previously explained DNA methylation is a dynamic epigenetic marker that is regulated by DNMT and TET enzymes that stimulate CpG methylation and demethylation respectively [49]. The importance of TET proteins and DNMTs in liver fibrosis was confirmed using a range of *in vivo* models of chronic liver disease (bile duct ligation (BDL), CCl_4_ and methionine-choline deficient (MCD) diet) [50]. Experimental liver fibrosis was accompanied by an induction of the *de novo* methyltransferases DNMT3A/B expression, diminution of TET protein expression and remodeling of the HSC DNA methylome. Moreover, culture activated HSC and *ex-vivo* purified HSC from CCl_4_ injured rats also demonstrated increased DNMT3A and DNMT3B, with knock-down of the latter enzymes resulting in reduced fibrogenic features. Of note, similar fibrosis-associated epigenetic changes were observed in mechanistically distinct examples of chronic human liver disease supporting the concept that fibrosis is in part driven by alterations in the balance between the DNMTs and TETs [51]. There is potential to target DNA methylation and this has been achieved in human cancers with 5-azadeoxycytidine (5-AzadC), a DNMT inhibitor [52], [53]. 5-AzadC is able to suppress HSC transdifferentiation providing further evidence of the importance of the DNA methylome in fibrosis [54]. However, given the likely need to treat chronic liver disease patients with anti-fibrotics for their lifetime it is difficult at present to envisage therapeutic manipulation of DNA methylation as a rationale strategy.

### 3.2 Histone modifications

Histone acetylation is associated with active transcription and is under the control of HATs and HDACs. Niki et al. first described the anti-fibrotic effects of HDAC inhibitors showing that trichostatin A (TSA) treatment of cultured HSC suppressed fibrogenic gene expression and proliferation [55]. HDACs are elevated in chronic liver disease and HDAC inhibitors have been shown to suppress HSC activation and proliferation [55], [56] leading to a suppression of fibrosis in numerous experimental models including *Schistosoma mansoni* and BDL induced liver fibrosis [57], [58]. However the specificity of these inhibitors is ill-defined and their functions are not completely understood. Therefore it is important to further study the activities and targets of currently available inhibitors and to generate more specific HDAC inhibitors for future investigation. Our laboratory described a role for HDAC1 as an epigenetic co-repressor of NF-κB p50:p50 mediated dampening of inflammatory and fibrogenic gene transcription in HSC [59]. More recently we showed that aged mice lacking p50 succumb to spontaneous fibrosis [60]. Hence, HDACs may have fibrosis promoting and inhibiting activities which complicates their therapeutic targeting. As previously discussed, environmental factors can induce the activity of histone modifiers. In 2005 Kim et al. demonstrated that ethanol exposure caused a dose and time dependent increase in acetylation of histone H3 at Lys9 in rat HSCs [61]. The H3K4 methyltransferase MLL1 is up-regulated in HSC transdifferentiated in the presence of ethanol, this leading to widespread changes in chromatin structure and altered expression of profibrogenic genes including elastin [62]. Targeting individual histone methyltransferases may be attractive and these enzymes are currently the subject of intense drug discovery [14]. However, they tend to be ubiquitous enzymes playing important functions in multiple cell types including hepatocytes. We and others have recently provided early proof-of-concept that selective *in vivo* delivery of histone methyltransferase inhibitors to HSC can potently inhibit liver fibrosis [43], [54], [63]. By encapsulating 3-deazaneplanocin A (dZNep), a broad specificity histone methyltransferase inhibitor, into liposomes coated with the HSC-specific single chain antibody C1–3, we were able to suppress HSC transdifferentiation and progressive fibrosis in the context of continuous CCl_4_-induced liver damage [64]. The BET histone readers are now emerging as potential targets for development of anti-fibrotics, as an example Ding et al. described BRD4 binding at the enhancers of fibrogenic genes and presented data suggesting that the BRD4 inhibitor JQ1 may be able to suppress or even reverse fibrosis *in vivo* [65].

### 3.3 Regulatory RNAs

A large number of miRNAs have been described that both promote (e.g. miR-145, miR-200a) and suppress (e.g. miR-338-3p, miR-378a-3p) HSC transdifferentiation [66], [67], [68], [69]. The most extensively characterised miRNAs in liver fibrosis are the miR-29 family [70]. Roderburg and colleagues discovered that all three members of the miR-29 family were downregulated in the liver of mice after CCl_4_ exposure or BDL, this observation being confirmed in human liver with advanced fibrosis [71]. Down-regulation of miR-29 in murine HSCs was mediated by TGF-β and experimental overexpression of miR-29b resulted in down-regulation of collagen expression [71]. More recently miR-29a overexpression was shown to ameliorate cholestatic liver fibrosis after bile duct ligation by decreasing HSC fibrotic gene expression, proliferation and migration [72]. In a subsequent study it was proposed that miR-29a mode of action was through the suppression of methyltransferases including DNMT1, DNMT3B and SET domain containing 1A (SET1A) leading to a DNA hypomethylation state that decreases fibrogenic activities in HSC [73]. Of note is that miR-29 mimicry (developed by miRagen Therapeutics) has been proposed as a therapy for pulmonary fibrosis [74]. A recent study by Zhou et al. has identified > 3600 lncRNAs in human HSC, many of which are regulated by TGF-β and enriched in extracellular matrix (ECM) networks. Significantly the authors go on to show that 16 lncRNAs that form a network with ECM proteins in adult HSC were also significantly enriched in fibrotic human liver providing *in vivo* relevance [75]. Homeobox transcript antisense RNA (HOTAIR), a long intergenic non-coding RNA, is upregulated in HSCs *in vivo* and *in vitro* during liver fibrosis, with HOTAIR knockdown suppressing HSC activation. The authors further demonstrated that HOTAIR downregulates miR-29b expression, attenuating its control on epigenetic regulation, leading to enhanced phosphatase and tensin homolog (PTEN) methylation, which contributes to the progression of liver fibrosis [76]. It is therefore highly probable that lncRNAs are playing an important role in HSC activation and function in the context of liver disease, however we are a considerable way from lncRNAs being exploited therapeutically or as fibrosis biomarkers.

## 4 Epigenetic drivers of hepatocellular carcinoma

Hepatocellular carcinoma (HCC) is the 3rd leading cause of cancer deaths worldwide and treatment options are severely limited. There is a clear need for the identification of the molecular drivers of HCC if progress is to be made in developing new medicines. Deep sequencing studies have been instrumental in identifying gene mutations and potential disease drivers in HCC. Alongside these genetic approaches is an increasing awareness of the role of epigenetic regulators in HCC which have strong potential for drug and biomarker design.

### 4.1 DNA methylation

DNA methylation changes are common in human cancers and it was therefore unsurprising that 3700 hypomethylated promoters were identified in HCC tumour samples [77]. Further analysis revealed the affected genes to be predominantly involved in cell proliferation, adhesion, cell signaling, mobility and invasion (i.e. ARF1, CASD1, MAP3K4, MMP14 and RALA) [78]. In contrast to these hypomethylated genes, a number of tumour suppressor genes (TSG) have been found to be hypermethylated in early HCC including HIC1, GSTP1, SOCS1, RASSF1, CDKN2A, APC, RUNX3 and PRDM2 [79], [80]. However, the caveat with all DNA methylation studies is that promoter methylation should be correlated with gene expression profiling to confirm that the modification is associated with a dramatic change in transcription. A recent study correlated 3 genome wide DNA methylation data sets (approx. 800 samples consisting of 646 tumour and 134 non tumour samples) with corresponding gene expression data sets. Results confirmed that hypermethylated patterns were highly consistent, with 84 sites from 61 promoters being hypermethylated in all 3 data sets including the previously reported CDKL2, TBX15 and NKX6-2 promoters. These data were used to subsequently classify tumour v non-tumour samples based on 10 selected probes. Integrative analysis identified 222 candidate epidrivers including the high confidence candidates SFN, SPP1 and TKT all significantly associated with patient overall survival [81].

There is vast potential for alterations in DNA methylation in HCC to be exploited for future drug and biomarker development. The de-methylating agent decitabine has been used to validate the expression of hypermethylated TSG in primary HCC and also re-expression of genes in HCC cell lines. 13 candidate TSG were identified in this study including DGK1, LDHB, NEFH, SMPD3, ACTL6B and PRPH. Functional characterisation of 2 candidates, SMPD3 and NEFH, revealed that overexpression leads to inhibition of cell proliferation, whereas by contrast knockdown increased tumour formation *in vivo*. Reduced SMPD3 expression was associated with early HCC recurrence following resection [82]. SMPD3 encodes an enzyme responsible for the production of ceramide in response to cellular stress [83]. It was recently reported that ceramide is markedly reduced in HCC tissue, hence epigenetic unmasking of SMPD3 may be of therapeutic value [84]. Guadecitabine, a dinucleotide anti-metabolite of decitabine, inhibits DNMTs and has been recently shown to suppress tumour growth *in vivo* in a xenograft HCC HepG2 model. CDKN2A, DLEC1 and RUNX3 promoters were all confirmed to be demethylated in HCC lines following Guadecitabine treatment and correlated with inhibition of cell growth. Importantly, in contrast to decitabine, Guadecitabine was able to overcome the inhibitory effects on cell growth by macroH2A1 (variant of histone H2A), highlighting Guadecitabine as a potential HCC therapeutic, particularly in advanced disease [85]. There is an urgent need for improved minimal invasive biomarkers for HCC, with measurement of serum alpha-fetoprotein (AFP) currently being the most commonly used marker for detection of HCC suffering from low sensitivity and specificity. DNA methylation can be readily detected and quantified in cell free circulating DNA and appears to reliably inform its tissue-of-origin [86]. Data are now beginning to emerge that suggest that detection of alterations in methylation in circulating cell free DNA in HCC patient plasma may offer a more sensitive biomarker platform [87].

### 4.2 Histone modifications

Histone modifying enzymes offer much promise for HCC therapy since they are tractable targets for the design of small molecule inhibitors. The H3K27 methylase EZH2 is up-regulated in HCC tissue and is associated with cancer progression, invasion and proliferation [88]. Animal studies have shown intratumoral knockdown of EZH2 promotes tumour regression, this observation being confirmed in HCC cell lines which lose their tumour initiating properties in the absence of EZH2 [89]. Mechanisms by which EZH2 promotes HCC are beginning to be revealed. Gao and colleagues have described a network of EZH2-regulated genes including CDKN2A, FOXO3, E2F1 and NOTCH2 that are silenced by EZH2 in HCC [90]. EZH2 silences multiple miRs identified as tumour suppressors [89]. Liu and colleagues have described how EZH2 represses the expression of miR-622 which is an important negative regulator of the chemokine receptor CXCR4 that is implicated in multiple steps of cancer development [91]. Loss of miR-622 was associated with elevated CXCR4 expression and poor HCC prognosis. EZH2 can also repress the expression of antagonists of the Wnt pathway thereby promoting β-catenin-dependent carcinogenesis. DZNEP is a potential therapy targeting polycomb proteins including EZH2. However, more specific EZH2 inhibitors including EPZ005687 and EPZ011989 are now available and have proved very successful at killing lymphoma cells, these new drugs may show therapeutic promise in EZH2 high HCC patients [92], [93]. As EZH2 is a good potential biomarker for HCC progression this may also enable easier monitoring and patient selection for anti-EZH2 therapy (Fig. 1). PR-SET7/SETD8 is the sole H4K20 methyltransferase and is a critical regulator of DNA repair and genome integrity [94], [95]. Nikolaou et al. reported that deletion of PR-SET7/SETD8 in mice results in spontaneous hepatocyte cell death, inflammation, fibrosis and cancer [96]. As PR-SET7/SETD8 is reported to be dysregulated in human HCC this may be a very relevant observation [97]. Recurrence rates for HCC are higher in patients with elevated expression of the H3K9 methyltransferase SUV39H1 and knockdown of this epigenetic writer impairs HCC cell growth [98]. Recruitment of HDACs to gene promoters results in loss of histone acetylation, compaction of chromatin and repression of transcription, a typical example is the p21 TSG which is transcriptionally repressed by multiple HDACs [99]. In addition, HDACs can repress transcription by removing acetyl modifications from many cancer-associated transcription factors including p53 and NF-κB [100]. Small molecule HDAC inhibitors have potent anticancer properties and several including vorinostat, romidepsin and belinostat are approved for immunological cancers [101]. In HCC, HDACs are able to promote silencing of TSGs such as CDHL1 and enhance cancer cell survival both *in vitro* and *in vivo* [102]. HDAC8 is upregulated in NAFLD-associated HCC and was reported to physically associate with EZH2 to bring about repression of Wnt antagonists and stimulate β-catenin to promote hepatocellular growth [103]. The NAD +-dependent HDAC Sirtuin 1 which regulates lipid, glucose and bile acid metabolism is over-expressed in HCC and promotes liver cancer at least in part via stimulating deactylation of the FXR and in turn dysregulation of bile acid homeostatis [104]. This latter study cautions against the use of sirtuin activating drugs. Clinical data to support the tumour-promoting role of HDACs include the overexpression of HDAC3 in 30–50% of HCC cases, particularly in HBV related HCC [105]. Additionally, overexpression of HDAC3 is associated with advanced tumour stage and early recurrence post-surgery [106]. HDAC1 overexpression is associated with high cancer cell invasion into the portal vein, poorer histological differentiation and more advanced tumour node metastasis as well as poor prognosis post resection [107]. Several inhibitors can efficiently inhibit HDAC activity in HCC cells including panobinostat, valproate and ITF2357 [102], [108]. Phase II trials with the HDAC inhibitor Belinostat in 42 patients with advanced unresectable HCC has demonstrated efficient tumour stabilization and is well tolerated [109]. However, progression free survival of 2.6 months and overall survival of 6.6 months does not improve on current therapy with Sorafenib [109]. Other HDAC inhibitors including abexinostat, resminostat, givinostat, panobinostat, pracinostat, vorinostat and CUDC-101 have shown encouraging anti-cancer properties at pre-clinical and clinical trials [110]. However, currently no HDAC inhibitors have shown any improvement on current sorafenib therapy for HCC.

### 4.3 Regulatory RNAs

Previous and emerging studies in HCC are now beginning to incorporate ncRNAs into our molecular understanding of HCC pathogenesis and progression [111]. The dysregulation of miRNA in cancer has been extensively reviewed [112]. Due to the stability of these small RNAs, sequencing from laser capture material, paraffin embedded tissue, exosomes and circulating serum is relatively robust. Hence, this has led to a wealth of RNA-seq data on dysregulated miRNA expression in tumour v non-tumour tissue in HCC [113]. miRNA from blood and tissue have been identified as prognostic indicators of HCC and used as biomarkers for early detection [113]. Viral proteins are potent modulators of various miRNA for example HbX which can downregulate the expression of miR-152 a known repressor of DMNT1, causing global hypermethylation including that of TSG promoters [114]. Recently, RNA sequencing from 23 liver tissues identified 5525 lncRNA, of which 57 were differentially expressed between tumour and adjacent non-tumour tissue and were co-expressed with genes involved in cell cycle control, TGFβ signaling and liver metabolism [115]. We have highlighted just a few lncRNA dysregulated in HCC (Table 1) however, new lncRNA are emerging every year including viral-human hybrid lncRNA such as HBx-(human) LINE1 linked to carcinoma progression [116].

**Table 1:** Dysregulated lncRNA in HCC.

Name	Biomarker	Function
**HOTAIR** (HOX transcript antisense intergenic RNA)	Upregulated in HCC and predictive of recurrence in transplant patients [120].	siRNA knockdown in HCC cell lines sensitizes to TNFα induced apoptosis, inhibits cell growth, induces cell cycle arrest and increases therapeutic sensitivity [120]. Recruits EZH2 to negatively regulate miR-218 expression subsequently resulting in the increased expression of the oncogene BMI-1 and inactivation of the tumour-suppressors p14ARF and P16Ink4a [121]. Inhibits the expression and phosphorylation of the methyltransferase SETD2 resulting in the reduction of H3K36me3 and subsequent formation of H3K36me3–hMSH2-hMSH6-SKP2 complexes, reduced DNA mismatch repair and the potential for microsatellite instability and abnormal expression of cell cycle related genes [122]. The viral protein HbX utilizes HOTAIR as a scaffold to induce proteasomal degradation of repressive chromatin regulators SUZ12 and ZNF198 leading to increased expression of cancer stem cell genes including EpCAM [123].
**MALAT1** (Metastasis-associated lung adenocarcinoma transcript 1)	Increased levels in 9 cancer cell lines and 112 HCC cases. High MALAT expression was significantly correlated with the increased risk of HCC recurrence post-transplant. Multivariate analysis demonstrates that MALAT1 is an independent prognostic factor for predicting HCC recurrence (hazard ratio, 3.280, P = 0.003) [124].	Inhibition in HEPG2 reduced viability, motility, invasiveness and increased sensitivity to apoptosis [124].
**HULC** (Highly up-regulated in liver cancer)	Expressed in normal hepatocytes and significantly increased in liver cancer (33 fold) [125].	A potential modulator of HBV- mediated hepatocarcinogenesis. HULC expression is up-regulated by HbX and shown to suppress the TSG p18 in HCC cell lines resulting in increased proliferation [126]. Promotes lipogenesis and proliferation in hepatoma cells. Shown to suppress miR-9 preventing its repression of PPARα and thereby promoting activation of acyl-CoA synthetase (ACSL1). The relevance of this effect was emphasised by the observation that cholesterol activated retinoid x receptor alpha (RXRA) further increased HULC levels [127]. Upregulates expression of the clock circadian regulator (CLOCK) resulting in increased proliferation of hepatoma cells both *in vitro* and *in vivo*[128]. HULC *via* SPHK1 plays a role in promoting angiogenesis by sequestering miR-107 and releasing its inhibition on E2F1 [129].
**HEIH** (Hepatocellular Carcinoma Up-Regulated EZH2-Associated Long Non-Coding RNA)	Increased HEIH expression in HBV-related HCC was significantly associated with HCC recurrence and is an independent prognostic factor for survival [130].	Knockdown of HEIH in HCC cells results in loss of repression of EZH2 target genes p16, p27, p21 resulting in G0/G1 arrest and reduced cell proliferation. The proliferative effects of HEIH are further supported by the observation that *in vivo* HEIH knockdown reduces xenograft growth [130].
**H19**	H19 is part of a conserved gene cluster containing the paternally expressed imprinted Igf2 gene [131]. Expression of both H19 and Igf2 are up-regulated in HBV-related HCC [128] and an imbalance between the proteins is associated with HCC progression [132].	Functionally, H19 promotes EZH2-mediated repression of E-cadherin, inhibitors of the WNT signaling pathway and enhances invasion and metastasis of HCC cell lines [133]. H19 reportedly plays a role in resistance to doxorubicin by promoter demethylation of MDR1 and subsequent expression of its protein P glycoprotein in HCC cell lines. Thus, suggesting that combination H19 targeting with chemotherapeutics may prove successful in cancer therapy [134].

As the complexity of these molecular interactions unravel, we can begin to appreciate that in most scenarios these modulators likely work together as a network of epigenetic regulators. This highlighted by a study demonstrating how HULC and MALAT1 combine in complex with TRF2 to significantly increase telomerase activity and microsatellite instability in liver cancer stem cells [117]. lncRNA can also act as molecular sponges inhibiting the function of tumour suppressor miRNA and influencing carcinogenesis. A deeper understanding of the mechanisms by which these lncRNA influence HCC initiation and progression will significantly advance the design of improved targeting therapies.

Therapeutic targeting of ncRNA in HCC is now an imminent challenge. Emerging pre-clinical studies utilizing sophisticated drug delivery nanoparticles and animal proof of concept models such as this study successfully targeting the lncRNA TUG1 show promise for therapeutic development of antisense oligonucleotides [118]. Clinical trials with the anti-miR-122 oligonucleotide Miravirsen have also shown promising effects in chronic HCV patients and demonstrate successful reduction in miR-122 levels [119]. Additionally, the development of MRG-201 (MIRagen Therapeutics) as a miR-29b agonist is currently in clinical trials for safety and efficacy with promise for liver fibrosis, if so it may also prove successful in advanced HCC (NCT02603224).

## 5 Summary and future prospective

The hepatic epigenome is remarkable in its capacity to adapt to dietary, metabolic, xenobiotic and microbial challenges in order to maintain cellular and functional homeostasis. However, when this capacity is breached epigenetic adaptions can become problematic and contribute to disease pathogenesis. We are now aware of how key components of the epigenetic machinery such as DNA methylation, histone PTMs and ncRNAs alter their expression or function in the context of fibrosis/cirrhosis and liver cancer. The next steps are to convert this information into bespoke medicines that either prevent disease-driving epigenome reprogramming or that reverse specific disease-promoting epigenetic changes. Furthermore there is vast potential to use the appearance of liver-derived epigenetic markers in the patient circulation for the diagnosis and prognostic tracking of chronic liver disease using no more than a few drops of blood, avoiding the need for biopsy or complex and expensive imaging modalities.
